# Quality assessment of a consultation-liaison psychiatry service

**DOI:** 10.1186/s12888-021-03281-4

**Published:** 2021-06-01

**Authors:** Zoltan Kovacs, Marton Asztalos, Simon Grøntved, René Ernst Nielsen

**Affiliations:** 1 Aalborg University Hospital, Psychiatry, Mølleparkvej 10, 9000 Aalborg, Denmark; 2 Department of Clinical Medicine, Aalborg University, Aalborg, Denmark; 3 Psychiatric Research Unit, North Denmark Region Psychiatry, Aalborg, Denmark

**Keywords:** Psychiatry, Referral and consultation, Quality of health care, Surveys and questionnaires, Comorbidity

## Abstract

**Background:**

Consultation-Liaison Psychiatry (CLP) provides services for patients with medical-psychiatric comorbidity at the general hospital. Referral satisfaction is considered as one of the most important outcome measures of CLP interventions. Our aim was to assess the levels of satisfaction with the CLP service amongst medical staff at a university hospital in Denmark.

**Methods:**

Medical staff answered an online survey regarding their experience with different aspects of inpatient and outpatient CLP services.

**Results:**

There were 152 responses from 16 medical units, with a survey return rate above 85%. Measured on a 5-point Likert scale, there was a median rating of 4 in response to questions regarding communication and organizational aspects, a median rating of 5 in response to questions regarding overall evaluation of the CLP service on both inpatient and outpatient questionnaire. The questions regarding treatment quality were rated with a median of 4 on the inpatient questionnaire and 2 of the outpatient questionnaire items, and with a median of 5 on 2 outpatient items. Physicians´ evaluations were statistically more positive than nurses´. As a group, respondents already employed before the CLP unit was established and those who used the CLP services more were statistically significantly more satisfied then respondents employed after the establishment of the CLP unit and those who used the CLP service less.

**Conclusion:**

The CLP services were positively appreciated and considered to be valuable among medical hospital staff. We believe that Consultation-Liaison Psychiatry deserves further help to implement and expand its services in general hospital settings. In addition, our results underline the feasibility of surveys as quality measures of clinical care.

**Supplementary Information:**

The online version contains supplementary material available at 10.1186/s12888-021-03281-4.

## Background

Individuals with mental disorders are more frequently affected by physical conditions [1, 2], whereas a large proportion of patients suffering from medical conditions also experience mental health problems [3–5]. Medical-psychiatric comorbidity is linked to increased length of hospital stay, higher medical costs and often rehospitalization [6]. Consultation-Liaison Psychiatry (CLP) is a subspecialty of psychiatry that provides assessment and treatment for general hospital patients with mental health comorbidities. It also provides teaching and research activities on mental health comorbidity to medical staff of non-psychiatric departments of general hospitals [7]. Although CLP services in the general hospital does not seem to reduce the length of hospitalization [8], they have a positive impact on mental health problems [9], and are cost effective [10]. Joint projects between medical and psychiatric professionals seem to ensure the best way to reduce the existing gap between medical specialties and psychiatry [11]; Consultation-Liaison Psychiatry services perform generally well in this context, with an earlier study showing an overall positive attitude towards CLP services among general medical hospital staff [12].

There is widespread agreement among professionals, managers and policy makers that an ongoing evaluation of CLP service quality using standardized measurements [13] is needed. Different measurement tools and quality indicators have been identified and developed in recent years, to assess consultee satisfaction [14], service utilization and efficiency [15], to secure quality improvement and accreditation [13], to improve service effectiveness [16] and to facilitate quality and outcome assessment and standardization of services [17]. However, a more widespread examination of CLP service quality across different countries [18], with an emphasis on using structured measurement tools [16] is needed. Since referrer satisfaction can be perceived as a proxy for a global measure of CLP service performance [19], it has been advocated that more attention should be paid to evaluating levels of staff satisfaction with different aspects of the CLP service [15].

Although staff feedback is recognized as important, there is a relative paucity of studies examining medical staff satisfaction with the CLP services. A review from 2014 identified only 13 studies on staff feedback between 1977 and 2011 [10], while in the more recent years only a few more studies have assessed consultee satisfaction [15, 20, 21]. Earlier studies solely focused on overall service evaluation rather than examining staff feedback on more specific aspects of the service [10]. To our knowledge no previous studies have assessed the impact of different consultee characteristics and service utilization parameters on satisfaction levels with CLP services.

The aim of the present study was to examine referrer satisfaction with the outpatient and inpatient CLP service across medical, surgical and ICU wards at a university hospital in Denmark, utilizing an online survey measuring predefined quality standards.

## Methods

### Setting

The CLP team consists of 2 psychiatrists, 2 nurses and one clinical psychologist. The team provides service for a 565-bed university hospital in Aalborg, Denmark, covering routine and urgent mental health assessments and follow-up during working hours for hospitalized medical patients, long-term outpatient follow-up after hospital discharge as well as outpatient assessment and treatment of patients referred from medical and surgical hospital outpatient departments. The CLP team provides outpatient and inpatient services for patients suffering from functional disorders, psychiatric symptoms and disorders associated with and resulting from medical diseases; psychiatric symptoms relating to pre-existing mental health disorders; assessment of suicidality and expert advice regarding psychopharmacological interventions in case of adverse medical events.

The psychiatric department at Aalborg University Hospital has a separate 24-h per day, 7-days per week psychiatric service responding to acute referrals from the general hospital wards and primary care services, as well as urgent referrals from hospital departments outside working hours and during weekends and holidays.

### Survey

Referrers’ experience and satisfaction was measured by a questionnaire regarding their experience with the outpatient and inpatient CLP service. The structure and content of the survey was inspired by similar examples [10, 13, 14], from which the relevant topics for measuring CLP were identified and described. The draft survey was sent to three senior physicians and two senior nurses for review and feedback to gather the perspectives of the general hospital staff. The survey was then tailored according to the questions and areas of interest they identified as being significant for measuring CLP performance. The final version of the survey contained 11 items assessing referrer satisfaction with a range of different aspects of the CLP service for the outpatient CLP service and 12 items for the inpatient CLP service (*Appendix 1*). The level of the responders agreement with the items was largely measured on a 5-point Likert scale ranging from “not-at-all” to “very much”. However one item “The extent of the CLP service” was rated utilizing a 3-point Likert scale ranging from “very unsatisfactory”, “unsatisfactory” to “appropriate”, while item “Overall evaluation of the CLP service” was rated on a 5 point Likert scale ranging from “very bad” to “very good”.

### Participants

Units receiving CLP services at a magnitude of at least 15 inpatients and 5 outpatients the preceding year were selected. Leading physicians and nurses at the respective units were asked to identify physicians and nurses who were users of the CLP service. More respondents were contacted at medical units with a higher number of CLP contacts. This was to ensure that the respondents had sufficient experience with and knowledge of the CLP service to make a valid evaluation and to include as many general hospital departments as possible. For general hospital wards with lower CLP contact numbers, the goal was to identify 3 physicians and 5 nurses as respondents, while for those with higher contact numbers 5 physicians and 6 nurses. The number of participants from the wards and outpatient units was chosen arbitrary by the authors.

An online survey tool (SurveyXact) was used to host the survey, to distribute the questionnaires and to collect results. The potential respondents were contacted via e-mail explaining the scope of the study, how data will be collected and stored and containing links to the online surveys. The responses were anonymous. In case of nonresponse, two reminder e-mails were sent with a one-week interval. The survey was performed between 1. November – 18. December 2019.

### Statistical analysis

As data is on an ordinal scale, we presented grouped answers as counts and percentages, and as medians with interquartile range (IQR).

For all comparisons of two groups (physicians versus nurses; respondents from wards with both inpatient and outpatient CLP referrals versus respondents from wards with only inpatient referrals and respondents working already before versus responders employed after setting up the CLP unit) we, for each survey-question, tested the hypothesis that the distribution is the same in both groups using Fisher’s exact test. For comparisons of three groups (healthcare professionals being in contact with 0–5 patients versus 6–10 patients versus more than 10 patients assessed by the CLP team) we, for each survey-question, tested the hypothesis that the distribution is the same in all groups using Fisher’s exact test.

When a user answered “I do not know”, that answer was not included in the final analyses. All counts of *n* = 1 or *n* = 2 were masked in the presented tables to ensure anonymity of all answers given.

## Results

A total of 16 general hospital units at Aalborg University Hospital were included in the survey. They consisted of 11 medical, 3 surgical and 3 ICU wards. All 16 units received inpatient service, and 7 of them had the opportunity to use, and did use, both the inpatient and outpatient services of the CLP unit. Potential respondents from these 7 units were given 2 questionnaires, addressing satisfaction with both the inpatient and outpatient service (i.e. inpatient and outpatient surveys), while only the inpatient survey was distributed to respondents from all 16 units.

For general hospital wards with lower CLP contact numbers, the goal was to identify 3 physicians and 5 nurses as respondents, for those wards with higher CLP contact numbers the goal was for 5 physicians and 6 nurses (though some wards could not fulfill this requirement).

Altogether there were 152 responses (overall response rate: 85,9%), 111 on the inpatient questionnaire (response rate: 82,8%) and 41 on the outpatient questionnaire (response rate: 95,3%). For a detailed description of the responders’ characteristics see Table 1.

**Table 1:** **Sample characteristics**

**Response rate**		
	**Sent surveys, N**	**Responses, N (%)**
Inpatient survey	134	111 (82.8%)
Outpatient survey	43	41 (95,3%)
**Hospital Units**		
	**Inpatient survey, N (%)**	**Outpatient survey, N (%)**
Anesthesiology and Intensiv Care Unit	6	
Cardiology	8	8
Cardiothoracic Surgery	5	
Emergency Medicin	7	
Endocrinology	4	7
Gastroenterology	8	5
Gastrointestinal Surgery	9	
Geriatrics	< 3	
Hematology	8	
Infectious Diseases	9	5
Nephrology	14	5
Neurology	8	5
Oncology	3	
Orthopaedic Surgery	5	
Pulmonology	11	6
Thoracic Intensiv Care Unit	< 3	
NA	3	
**Responder characteristics**		
	**Inpatient survey, N (%)**	**Outpatient survey, N (%)**
Physicians	53 **(48)**	34 **(83)**
Working already before 7	70 **(63)**	33 **(80)**
Referred cases to CLP		
0–5 cases	31 **(28)**	5 **(12)**
6–10 cases	25 **(23)**	16 **(39)**
More than 10 cases	55 **(50)**	20 **(49)**

Overall, the answers showed a high satisfaction level with both inpatient and outpatient CLP services. For the inpatient questionnaire, 8 items received a median rating of 4 (“agree much” with the statement), and 4 items received a median rating of 5 (“agree very much” with the statement) on a 5-point Likert scale. For the outpatient questionnaire, 5 items received a median rating of 4, and 6 items received a median rating of 5 (Table 2). We have divided the items of the survey into 4 subgroups (see Appendix 1): Organizational aspects, Communication, Treatment quality and Overall evaluation. Respondents to both the inpatient and outpatient survey gave a median rating of 5 to questions measuring “Overall evaluation”, and a median rating of 4 to questions measuring “Communication” and “Organizational aspects”. Regarding “Treatment Quality”, respondents to the outpatient survey gave a median rating of 4 to 2 questions and a median rating of 5 to the other 2 questions, while respondents to the inpatient survey gave a median rating of 4 to all questions.

**Table 2:** Satisfaction with the CLP service

	To a very large degree				To a large degree				To some degree				Low degree or Not at all				p						
	Inpatient		Outpatient		Inpatient		Outpatient		Inpatient		Outpatient		Inpatient		Outpatient			Inpatient			Outpatient		
	n	%	n	%	n	%	n	%	n	%	n	%	n	%	n	%		1qrt	median	3qrt	1qrt	median	3qrt
Referral process	6	**7**	7	**18**	45	**52**	22	**55**	28	**33**	9	**22**	7	**8**	< 3	**NA**	0,025	4,00	**4,00**	3,00	4,00	**4,00**	3,50
Relevant timeframe	34	**34**	18	**49**	57	**56**	19	**51**	7	**7**	0	**0**	3	**3**	0	**0**	0,410	5,00	**4,00**	4,00	5,00	**4,00**	4,00
Accessibility	19	**21**	14	**38**	46	**51**	18	**49**	22	**24**	3	**8**	3	**3**	< 3	**NA**	0,121	4,00	**4,00**	3,00	5,00	**4,00**	4,00
Information provided after the assessment	44	**42**			47	**45**			9	**9**			5	**5**				5,00	**4,00**	4,00			
Matching the referrer’s or patient’s needs	26	**28**	17	**47**	41	**44**	17	**47**	25	**27**	< 3	**NA**	< 3	**NA**	0	**0**	0,051	5,00	**4,00**	3,00	5,00	**4,00**	4,00
Improved mental state	28	**29**	18	**44**	40	**42**	21	**51**	26	**27**	< 3	**NA**	< 3	**NA**	0	**0**	0,000	5,00	**4,00**	3,00	5,00	**4,00**	4,00
Improved compliance	26	**25**	21	**54**	52	**51**	15	**38**	21	**21**	3	**8**	3	**3**	0	**0**	0,036	4,75	**4,00**	4,00	5,00	**5,00**	4,00
Ease of treatment	37	**36**	23	**61**	41	**39**	12	**32**	23	**22**	3	**8**	3	**3**	0	**0**	0,103	5,00	**4,00**	3,75	5,00	**5,00**	4,00
Service quality	53	**60**	31	**78**	27	**30**	9	**22**	7	**8**	0	**0**	< 3	**NA**	0	**0**	0,002	5,00	**5,00**	4,00	5,00	**5,00**	5,00
Referrers perceived need	69	**67**	35	**83**	29	**28**	5	**12**	5	**5**	< 3	**NA**	0	**0**	0	**0**	0,035	5,00	**5,00**	4,00	5,00	**5,00**	5,00
Overall satisfaction	62	**58**	36	**88**	39	**36**	5	**12**	4	**4**	0	**0**	< 3	**NA**	0	**0**	0,006	5,00	**5,00**	4,00	5,00	**5,00**	5,00
	Adequate								Unsatisfactory				Very unsatisfactory										
Perceived extent	86	**85**	34	**87**					13	**13**	5	**13**	< 3	**< 3**	0	0	0,787	5,00	**5,00**	4,00	5,00	**5,00**	5,00

1 = Not at all.

2 = Low degree.

3 = To some degree.

4 = To a large degree.

5 = To a very large degree.

We also examined any potential differences to responses on satisfaction between the different subgroups.

There were statistically significant differences in responses on all but two questions between physicians and nurses showing that physicians were more satisfied overall with the communication with the CLP team, the treatment quality provided by the CLP service, some aspects of the organization of the CLP service and expressed a higher satisfaction with the CLP service in general (Table 3).

**Table 3:** Differences in satisfaction between physicians and nurses

	To a very large degree				To a large degree				To some degree				Low degree or Not at all				p						
	Physicians		Nurses		Physicians		Nurses		Physicians		Nurses		Physicians		Nurses			Physicians			Nurse		
	n	%	n	%	n	%	n	%	n	%	n	%	n	%	n	%		1qrt	median	3qrt	1qrt	median	3qrt
Referral process	12	**15**	< 3	**NA**	46	**58**	20	**43**	18	**23**	19	**41**	3	**4**	5	**11**	< 0.001	4,00	**4,00**	3,00	4,00	**3,00**	3,00
Relevant timeframe	38	**46**	14	**25**	43	**52**	32	**58**	0	**0**	7	**13**	< 3	**NA**	< 3	**NA**	< 0.001	5,00	**4,00**	4,00	4,50	**4,00**	4,00
Accessibility	28	**36**	5	**11**	37	**47**	26	**55**	11	**14**	14	**30**	< 3	**NA**	< 3	**NA**	< 0.001	5,00	**4,00**	4,00	4,00	**4,00**	3,00
Information provided after the assessment	26	**50**	18	**35**	22	**42**	24	**46**	< 3	**NA**	7	**13**	< 3	**NA**	3	**6**	0,100	5,00	**4,50**	4,00	5,00	**4,00**	4,00
Matching the referrer’s or patient’s needs	36	**43**	7	**14**	37	**45**	21	**43**	8	**10**	19	**39**	< 3	**NA**	< 3	**NA**	< 0.001	5,00	**4,00**	4,00	4,00	**4,00**	3,00
Improved mental state	35	**43**	11	**20**	41	**50**	19	**35**	4	**5**	23	**42**	< 3	**NA**	< 3	**NA**	< 0.001	5,00	**4,00**	4,00	4,00	**4,00**	3,00
Improved compliance	37	**44**	10	**18**	41	**48**	25	**45**	5	**6**	19	**34**	< 3	**NA**	< 3	**NA**	< 0.001	5,00	**4,00**	4,00	4,00	**4,00**	3,00
Ease of treatment	46	**54**	14	**25**	28	**33**	25	**44**	9	**11**	16	**28**	< 3	**NA**	< 3	**NA**	< 0.001	5,00	**5,00**	4,00	4,00	**4,00**	3,00
Service quality	62	**75**	22	**49**	17	**20**	18	**40**	< 3	**NA**	5	**11**	< 3	**NA**	0	**0**	< 0.001	5,00	**5,00**	5,00	5,00	**4,00**	4,00
Referrers perceived need	72	**84**	31	**54**	12	**14**	22	**39**	< 3	**NA**	4	**7**	0	**0**	0	**0**	< 0.001	5,00	**5,00**	5,00	5,00	**5,00**	4,00
Overall satisfaction	72	**82**	25	**40**	12	**14**	32	**52**	< 3	**NA**	3	**5**	< 3	**NA**	< 3	**NA**	< 0.001	5,00	**5,00**	5,00	5,00	**4,00**	4,00
	Adequate								Unsatisfactory				Very unsatisfactory										
Perceived extent	74	**89**	45	**83**					9	**11**	9	**17**	0	**0**	0	**0**	0,062	5,00	**5,00**	5,00	5,00	**5,00**	4,00

1 = Not at all.

2 = Low degree.

3 = To some degree.

4 = To a large degree.

5 = To a very large degree.

There were no statistically significant differences between assessments from respondents at medical units receiving both outpatient and inpatient service and those receiving only inpatient service, except on one item showing that teams who utilized both inpatient and outpatient CLP services were more satisfied with the communication with the CLP team (Table 4).

**Table 4:** Differences in satisfaction among responses from units receiving both inpatient and outpatient CLP services vs. units only receiving inpatient service

	To a very large degree				To a large degree				To some degree				Low degree or Not at all				p						
	Received service				Received service				Received service				Received service					In-& outpatient			Only inpatient		
	In-&outpatient		Only inpatient		In-&outpatient		Only inpatient		In-&outpatient		Only inpatient		In-&outpatient		Only inpatient								
	n	%	N	%	n	%	n	%	n	%	n	%	n	%	n	%		1qrt	median	3qrt	1qrt	median	3qrt
Referral process	4	**8**	< 3	**NA**	26	**52**	19	**53**	17	**34**	11	**31**	3	**6**	4	**11**	0,610	4,00	**4,00**	3,00	4,00	**4,00**	3,00
Relevant timeframe	21	**37**	13	**30**	34	**60**	23	**52**	< 3	**NA**	5	**11**	0	**NA**	3	**7**	0,084	5,00	**4,00**	4,00	5,00	**4,00**	4,00
Accessibility	13	**26**	6	**15**	27	**54**	19	**48**	10	**20**	12	**30**	0	**0**	3	**8**	0,258	4,75	**4,00**	4,00	4,00	**4,00**	3,00
Information provided after the assessment	28	**47**	16	**35**	28	**47**	19	**41**	< 3	**NA**	7	**15**	< 3	**< 6%**	4	**9**	0,025	5,00	**4,00**	4,00	5,00	**4,00**	4,00
Matching the referrer’s or patient’s needs	17	**31**	9	**22**	23	**42**	18	**44**	13	**24**	12	**29**	< 3	**4**	< 3	**NA**	0,285	5,00	**4,00**	3,25	4,00	**4,00**	3,00
Improved mental state	17	**31**	11	**26**	22	**41**	18	**43**	15	**28**	11	**26**	0	**0**	< 3	**NA**	0,548	5,00	**4,00**	3,00	4,75	**4,00**	3,00
Improved compliance	16	**27**	10	**23**	29	**49**	23	**52**	12	**20**	9	**20**	< 3	**NA**	< 3	**NA**	0,309	5,00	**4,00**	4,00	4,00	**4,00**	3,75
Ease of treatment	22	**38**	15	**33**	22	**38**	19	**41**	14	**24**	9	**20**	0	**0**	3	**7**	0,295	5,00	**4,00**	4,00	5,00	**4,00**	3,25
Service quality	34	**69**	19	**48**	11	**22**	16	**40**	4	**8**	3	**8**	0	**0**	< 3	**5**	0,199	5,00	**5,00**	4,00	5,00	**4,00**	4,00
Referrers perceived need	41	**72**	28	**60**	14	**25**	15	**32**	< 3	**NA**	4	**9**	0	**0**	0	**0**	0,326	5,00	**5,00**	4,00	5,00	**5,00**	4,00
Overall satisfaction	37	**62**	25	**53**	21	**35**	18	**38**	< 3	**NA**	< 3	**NA**	0	**0**	< 3	**NA**	0,109	5,00	**5,00**	4,00	5,00	**5,00**	4,00
	Adequate								Unsatisfactory				Very unsatisfactory										
Perceived extent	49	**89**	37	**80**					4	**7**	9	**20**	2	4	0	0	0,251	5,00	**5,00**	4,00	5,00	**5,00**	4,00

1 = Not at all.

2 = Low degree.

3 = To some degree.

4 = To a large degree.

5 = To a very large degree.

There were statistically significant differences in evaluations on all but three questions between healthcare professionals who were already working at their respective medical units before the CLP unit was established and those who started working afterwards with data showing referrers who were working before the CLP service was set up were more satisfied with some aspects of the organization of the CLP service and the communication with the CLP team, and more satisfied overall with the treatment quality provided by the CLP service and expressed a higher satisfaction with the CLP service in general (Table 5).

**Table 5:** Differences in satisfaction between healthcare professionals already working before versus employed after the CLP service was established

	To a very large degree				To a large degree				To some degree				Low degree or Not at all				p						
	Employed				Employed				Employed				Employed					Employed before			Employed after		
	Before		After		Before		After		Before		After		Before		After								
	n	%	n	%	n	%	n	%	n	%	n	%	n	%	n	%		1qrt	median	3qrt	1qrt	median	3qrt
Referral process	11	**13**	< 3	**NA**	47	**55**	20	**51**	22	**26**	15	**38**	6	**7**	< 3	**NA**	0,501	4,00	**4,00**	3,00	4,00	**4,00**	3,00
Relevant timeframe	41	**45**	11	**23**	45	**49**	31	**66**	4	**4**	3	**6**	< 3	**NA**	< 3	**NA**	0,036	5,00	**4,00**	4,00	4,00	**4,00**	4,00
Accessibility	27	**32**	6	**15**	40	**47**	24	**59**	16	**19**	9	**22**	< 3	**NA**	< 3	**NA**	0,247	5,00	**4,00**	4,00	4,00	**4,00**	3,00
Information provided after the assessment	32	**49**	12	**30**	29	**45**	18	**45**	4	**6**	5	**12**	0	**0**	5	**12**	0,019	5,00	**4,00**	4,00	5,00	**4,00**	3,75
Matching the referrer’s or patient’s needs	35	**40**	8	**19**	39	**45**	19	**44**	13	**15**	14	**33**	0	**0**	2	**NA**	0,016	5,00	**4,00**	4,00	4,00	**4,00**	3,00
Improved mental state	34	**37**	12	**27**	46	**50**	15	**34**	12	**13**	15	**34**	0	**0**	2	**NA**	0,014	5,00	**4,00**	4,00	5,00	**4,00**	3,00
Improved compliance	36	**38**	11	**24**	47	**49**	20	**43**	12	**13**	12	**26**	0	**0**	3	**7**	0,035	5,00	**4,00**	4,00	4,00	**4,00**	3,00
Ease of treatment	46	**48**	14	**30**	39	**41**	14	**30**	10	**11**	16	**34**	0	**0**	3	**6**	0,001	5,00	**4,00**	4,00	5,00	**4,00**	3,00
Service quality	70	**74**	14	**40**	21	**22**	15	**43**	3	**3**	4	**11**	0	**0**	< 3	**NA**	< 0.001	5,00	**5,00**	4,25	5,00	**4,00**	4,00
Referrers perceived need	78	**77**	26	**60**	20	**20**	14	**33**	3	**3**	3	**7**	0	**0**	0	**0**	0,008	5,00	**5,00**	5,00	5,00	**5,00**	4,00
Overall satisfaction	76	**75**	22	**46**	23	**23**	21	**44**	< 3	**NA**	3	**6**	0	**0**	< 3	**NA**	0,001	5,00	**5,00**	5,00	5,00	**4,00**	4,00
	Adequate								Unsatisfactory				Very unsatisfactory										
Perceived extent	83	**86**	37	**84**					13	**14**	5	**11**	0	**0**	< 3	**NA**	0,336	5,00	**5,00**	5,00	5,00	**5,00**	5,00

1 = Not at all.

2 = Low degree.

3 = To some degree.

4 = To a large degree.

5 = To a very large degree.

There were statistically significant differences on all but one question in the responses from subjects who had more patients assessed and treated by the CLP team compared to responses from those subjects who had fewer patients in contact with the CLP team with data showing referrers who utilized the CLP team more were more satisfied overall with the organization of the CLP service, the communication with the CLP team, the treatment quality provided by the CLP service and expressed a higher satisfaction with the CLP service in general (Table 6).

**Table 6:** Differences in satisfaction between groups divided on the basis of their number of patients assessed and treated by the CLP unit

	To a very large degree						To a large degree						To some degree						Low degree or Not at all						p									
	Patients, N						Patients, N						Patients, N						Patients, N							Patients, N			Patients, N			Patients, N		
	0–5		6–10		> 10		0–5		6–10		> 10		0–5		6–10		> 10		0–5		6–10		> 10			0–5			6–10			> 10		
	n	%	n	%	n	%	n	%	n	%	n	%	n	%	n	%	n	%	n	%	n	%	n	%		1qrt	median	3qrt	1qrt	median	3qrt	1qrt	median	3qrt
Referral process	< 3	**NA**	3	**8**	8	**12**	7	**35**	16	**41**	44	**66**	9	**45**	18	**46**	10	**15**	< 3	**NA**	< 3	**NA**	5	**7**	< 0.001	4,00	**3,00**	3,00	4,00	**3,50**	3,00	4,00	**4,00**	4,00
Relevant timeframe	5	**18**	15	**36**	32	**44**	19	**68**	23	**55**	34	**47**	< 3	**NA**	< 3	**NA**	4	**6**	< 3	**NA**	< 3	**NA**	< 3	**NA**	< 0.001	4,00	**4,00**	4,00	5,00	**4,00**	4,00	5,00	**4,00**	4,00
Accessibility	4	**14**	12	**33**	17	**27**	17	**61**	16	**44**	31	**48**	5	**18**	6	**17**	14	**22**	< 3	**NA**	< 3	**NA**	< 3	**NA**	0,509	4,00	**4,00**	4,00	5,00	**4,00**	4,00	5,00	**4,00**	3,75
Information provided after the assessment	3	**12**	12	**46**	29	**52**	13	**52**	12	**46**	22	**39**	6	**24**	0	**0**	3	**5**	< 3	**NA**	< 3	**NA**	< 3	**NA**	< 0.001	4,00	**4,00**	3,00	5,00	**4,00**	4,00	5,00	**5,00**	4,00
Matching the referrer’s or patient’s needs	4	**17**	12	**30**	27	**40**	13	**54**	22	**55**	23	**34**	5	**21**	6	**15**	16	**24**	< 3	**NA**	0	**0**	< 3	**NA**	0,001	4,00	**4,00**	3,50	5,00	**4,00**	4,00	5,00	**4,00**	3,50
Improved mental state	7	**26**	16	**40**	23	**32**	12	**44**	15	**38**	34	**48**	6	**22**	9	**22**	12	**17**	< 3	**NA**	0	**0**	< 3	**NA**	0,016	4,75	**4,00**	3,25	5,00	**4,00**	4,00	5,00	**4,00**	4,00
Improved compliance	5	**17**	14	**35**	28	**38**	16	**55**	21	**52**	30	**41**	6	**21**	5	**12**	13	**18**	< 3	**NA**	0	**0**	< 3	**NA**	0,009	4,00	**4,00**	3,75	5,00	**4,00**	4,00	5,00	**4,00**	4,00
Ease of treatment	8	**26**	19	**50**	33	**45**	9	**29**	16	**42**	28	**38**	12	**39**	3	**8**	11	**15**	< 3	**NA**	0	**0**	< 3	**NA**	0,001	4,75	**4,00**	3,00	5,00	**4,50**	4,00	5,00	**4,00**	4,00
Service quality	9	**33**	30	**79**	45	**70**	11	**41**	8	**21**	17	**27**	5	**19**	0	**0**	< 3	**NA**	< 3	**NA**	0	**0**	0	**0**	< 0.001	5,00	**4,00**	4,00	5,00	**5,00**	5,00	5,00	**5,00**	4,00
Referrers perceived need	14	**45**	35	**90**	55	**74**	15	**48**	4	**10**	15	**20**	< 3	**NA**	0	**0**	4	**5**	0	**0**	0	**0**	0	**0**	0,001	5,00	**4,00**	4,00	5,00	**5,00**	5,00	5,00	**5,00**	4,25
Overall satisfaction	13	**39**	31	**74**	54	**71**	15	**45**	9	**21**	20	**26**	< 3	**NA**	< 3	**NA**	0	**0**	< 3	**NA**	0	**0**	< 3	**NA**	< 0.001	5,00	**4,00**	4,00	5,00	**5,00**	5,00	5,00	**5,00**	4,00
	Adequate												Unsatisfactory						Very unsatisfactory															
Perceived extent	21	**81**	35	**88**	64	**86**							5	**19**	5	**13**	8	**10**	0	**0**	0	**0**	< 3	**NA**	0,001	5,00	**5,00**	2,00	5,00	**5,00**	5,00	5,00	**5,00**	5,00

1 = Not at all.

2 = Low degree.

3 = To some degree.

4 = To a large degree.

5 = To a very large degree.

At the end of the questionnaires, the respondents were able to add comments. The vast majority of these comments underlined the following: the important role that the CLP service plays; emphasized that CLP service gives a clear boost to the quality of treatment for their patients; highlighted the consultants’ professional, committed and friendly approach and cooperation skills; and praised the CLP service for educating staff at the general hospital. However, a few respondents mentioned some issues with the referral procedure (unclear referral process and criteria), expressed some dissatisfaction with the lack of CLP service during the weekends, and suggested that the service receive more promotion.

## Discussion

There is widespread agreement in the literature that medical staff satisfaction is one of the most important performance measures of CLP services. In order to assess the performance of our CLP unit, we investigated the subjective satisfaction with different aspects of the CLP service among a large representative sample of physicians and nurses from 16 different medical, surgical and ICU units at a university hospital.

Overall, the results showed high levels of satisfaction with inpatient and outpatient CLP services, including with specific aspects such as treatment and service quality, organization and communication. Respondents expressed strong levels of agreement that the CLP unit ensures a relevant assessment and treatment of medical patients with mental health problems, improves the patients´ mental state and compliance, resulting in greater benefits from the medical treatment and simultaneously making the treatment of these patients easier for the medical staff. There was also a strong agreement among respondents that the guidelines regarding referral processes to the CLP team were clear and patients were assessed within a relevant timeframe, that the CLP team could be easily contacted and that findings and results were communicated in an appropriate manner. Furthermore, respondents expressed very positive satisfaction levels overall and a very high perceived need for the CLP services. In addition they felt to a very large degree that the CLP unit provided a higher quality of service compared to psychiatric treatment as usual.

From a methodological point of view, because our study sample consisted of a representative group of physicians and nurses with an overall survey return rate above 85%, we believe our results can be generally applicable for medical staff at general hospitals as a whole. Furthermore, the present study underscores the feasibility of evaluating ongoing clinical services utilizing surveys as a measure.

Our work adds to the existing knowledge on the position and quality of CLP services in the general medical hospital. A widespread survey of consultation-liaison organizations in the Western European countries was conducted almost 30 years ago [22], and while more recently the CLP activities in three different countries (2 from Europe and 1 from Asia) were also discussed, the authors concluded that there was still a need for further research on describing the status of CLP in different countries [18]. A systematic review from 2014 [10] which included 13 studies examining staff feedback on CLP services, showed general satisfaction levels between 21 to 100%; the majority of studies reported satisfaction rates between 56 and 86%. A study from 2014 with 41 responses and a response proportion of 59% from consultants at a general hospital [15] showed overall satisfaction with the CLP service as being at 63 to 98% of staff members. Although slightly different rating scales were used, the levels of satisfaction studied were comparable with our results. However, follow-up arrangements and helping staff members' better manage mental health problems in the medically ill reported levels of satisfaction at a lower level than those found in our study regarding the inpatient CLP service. In addition, a study published in 2019 based on 170 responses from nurses and physicians at medical wards showed that increased levels of staff satisfaction with inpatient CL assessment were associated with providing confidence, support and improved communication amongst medical staff [21]. Among the Nordic countries, the nationwide organization and quality of services for CLP has been examined and described in Norway, results show a moderate level of satisfaction among medical staff with the inpatient CLP service (mean of 3.2 on a scale from 1-very poor to 5-very good) [20]. The qualitative evaluation of a 1-year project involving a psychiatric CLP nurse at a medical ward in Denmark [23] concluded that the project helped medical staff treating their patients with comorbid psychiatric problems more efficiently, achieving a better understanding of the mind-body connection and improving their communication skills. In the recent period, there has been a shift from focusing solely on functional disorders to a growing awareness of other areas of possible importance for the CLP services in Denmark [24], with Danish authorities now recognizing the existing unmet needs and pushing for a closer and more structured collaboration between the general hospital and psychiatry via establishing specialized CLP Units [25].

Nurses in our study were significantly less satisfied with every aspect of the CLP service than physicians. This finding stands in contrast with results from a previous study assessing referrer satisfaction with a telepsychiatric CLP service, showing that nursing staff rated the service more positively than general practitioners [26] as well as a more recent study which found no differences in how nurses and physicians respectively rated satisfaction with CLP service [21] There can be several explanations for the fact that our service is seemingly more suited to meet the demands of physicians than nurses. It has been shown that nurses and physicians have different perceptions of and demands from CLP services, with nurses expressing more need for on-the-spot verbal advice, and physicians having more emphasis on written clinical risk evaluations and overall management plans [27]. Referrals to our CLP team were always made by physicians, resulting in the CLP team’s assessment and the consequent management plan being more reflective of the physicians’ referral problem formulation and demands, rather than the nurses’, which were not necessarily directly expressed during the referral process, and thus might be unfamiliar to the CLP team. Although generally highly satisfied with the CLP service, this group possibly felt that their demands were not fully met. On the other hand, it is highly important for referrers from medical units that the CLP team understands the clinical situation as well as the scope of the referral [19]; when this does not happen as expected, the result can be a lesser degree of satisfaction with that particular service, in our case the CLP service. Furthermore, in our clinical experience, there is an implicit perception among nurses that the CLP team should help them cope with a wide range of compliance and behavioral problems, which does not necessarily lie inside the CLP competence, possibly resulting in a perception of unmet demands.

Respondents from units receiving both inpatient and outpatient CLP services were more satisfied with the information after the assessment; otherwise we could not identify further differences compared to those departments who only received CLP inpatient services.

Respondents who had more patients assessed and treated by the CLP team evaluated the service quality as significantly higher. This result is in line with other findings showing that positive attitude towards CLP among medical staff is strongly associated with the number of patients being referred [28]. It is on the other hand also plausible that simply referring more patients and hence gathering more knowledge of the scope and possibilities of the CLP service can result in more satisfaction with the service.

Furthermore, those referrers who were already working at their respective medical units before our CLP service was established were significantly more satisfied with almost all measured aspects of the CLP service, the most striking difference being a significantly more positive evaluation of the CLP service compared to psychiatric service as usual, e.g. on call psychiatrist or general psychiatric outpatient services. It seems plausible, that this finding can be related to the positive effects of the CLP team having a close link and working together with the general hospital staff towards common goals and in the meantime showing high professional standards, as supported by the comments on the questionnaires, which commended the committed and friendly attitude of the CLP team members. Indeed, close working relationships with other medical specialties and appropriate professional behavior of psychiatric team members are considered key factors in improving the existing negative image of psychiatry and closing the gap between psychiatry and medical specialties [11].

The collected responses and comments expressed high levels of satisfaction with the workings of the CLP team, which was described as exceptionally professional and highly effective from several different aspects. The responders emphasized that the CLP service provided the sort of assistance they had needed and lacked for a long time, it helped solving psychological and mental health problems that had so far remained untreated, shortened the treatment period, improved the patients’ ability to cope with their medical disorder, and raised their chances of recovery. Medical staff stressed that their professional competencies and satisfaction with their own work had also improved, in part because they gained a better psychiatric knowledge and understanding of psychological and psychiatric issues, and in part because of the added possibility of referring to the CLP team for assessment and treatment of conditions they had previously not been able to deal with on their own.

Our study has some limitations. Data was collected from a single hospital, and only a relatively modest proportion of medical staff were contacted. Furthermore, we only collected and analyzed data obtained via the survey from referrers to the CLP service, and did not assess other data-capture methods of potential interest for the assessment of CLP service quality (e.g. clinical data, patient reported outcomes etc.). Although the content and structure of our survey was inspired by parameters and standards already identified and outlined in literature, the questionnaire used has not been validated. The fact that surveys were only sent to members of medical staff identified by their respective leaders as users of the CLP services and being interested in answering the surveys, may have artificially inflated the results due to selection bias; furthermore half of the respondents were frequent users of the CLP service, which might further inflate the scores. The present study was designed to collect information on medical staff satisfaction with an already existing CLP service; the study was not designed to identify possibilities to strengthen the CLP service. Future studies should focus more on identifying potential areas of improvement for existing CLP services.

## Conclusions

The present study utilized survey data on referrers’ perceived quality of the Consultation-Liaison Psychiatric service from respondents representing a broad and part of general staff from medical, surgical and intensive care units at a university hospital. Data showed a high level of staff satisfaction with treatment and organizational aspects of the CLP service. The present study underscores the feasibility of evaluating ongoing clinical services utilizing surveys as a measure. We believe that our study further contributes to gathering valuable feedback on such an important outcome standard as referrer satisfaction and helping to implement and expand Consultation-Liaison Psychiatry.

## Supplementary Information

**Additional file 1.**

## Data Availability

The datasets used and/or analysed during the current study are available from the corresponding author on reasonable request.
