# Supplementary material for: Quality assessment of a consultation-liaison psychiatry service
Source: BMC Psychiatry. 2021 Jun 1;21:281. doi: 10.1186/s12888-021-03281-4 (PMC8167950; doi:10.1186/s12888-021-03281-4)
Supplement: Supplementary file 1 — Additional file 1. [file 12888_2021_3281_MOESM1_ESM.docx]

**Appendix 1. Questionnaires**

| **Short version** | **Inpatient questionnaire** | **Outpatient questionnaire** | **Question category** |
| --- | --- | --- | --- |
| Referral proces | The inpatient referral criteria for the elective psychiatric consultation are unambiguous. | The outpatient referral criteria to the CLP Unit are unambiguous. | *Organizational aspects* |
| Relevant timeframe | Patients who need elective consultation were seen within a relevant timeframe between request and assessment. | Patients who need outpatient follow-up were seen within a relevant timeframe between request and assessment. | *Organizational aspects* |
| Accessibility | The CLP Unit can easily be contacted when questions arise before/after the psychiatric consultation. | The CLP Unit can easily be contacted, when questions arise related to the referral or to the outpatient follow-up. | *Communication* |
| Information provided after the assessment | Relevant findings and treatment recommendations are clearly communicated to the ward after the elective psychiatric consultation. | - | *Communication* |
| Matching the referrer´s/patient´s need | If necessary, a follow-up consultation will be performed by the CLP Unit during hospitalization and a plan will be made for further outpatient treatment in the Primary Sector or Psychiatry. | Patients receive relevant outpatient treatment at the CLP Unit. Alternatively, the patient is referred to other relevant treatment options in Psychiatry. | *Treatment quality* |
| Improved mental state | The elective psychiatric consultation helps patients to achieve an improved state of mental health. | CLP`s outpatient service helps patients to achieve an improved state of mental health. | *Treatment quality* |
| Improved compliance | The elective psychiatric consultation ensures that patients with mental health problems in somatic wards can cooperate and benefit to a greater extent from the somatic treatment. | The outpatient CLP offer ensures that patients with mental health problems in somatic wards and outpatient units can cooperate and benefit to a greater extent from the somatic treatment. | *Treatment quality* |
| Ease of treatment | The elective psychiatric consultation makes it easier for somatic ward staff members to treat patients with mental health problems. | The outpatient CLP offer makes it easier for somatic ward staff members to treat patients with mental health problems. | *Treatment quality* |
| Service quality | CLP Unit has provided a quality boost compared to the psychiatric consultation ”as usual” (consultation via acute psychiatric service). | CLP Unit has provided a quality boost regarding the treatment of somatic patients with psychiatric problems compared to “treatment as usual” (referral to Psychiatry or follow up at General Practitioners). | *Overall evaluation* |
| Referrers perceived need | There is a perceived need for the CLP Unit`s inpatient consultations. | There is a perceived need for the CLP Unit`s outpatient examination- and treatment service. | *Overall evaluation* |
| Overall satisfaction | Overall impression of the elective psychiatric consultation. | Overall impression of the outpatient CLP service. | *Overall evaluation* |
| Perceived extent | The extent of the CLP Unit`s inpatient consultation offer compared to the needs of the ward. | The extent of the CLP Unit`s outpatient service offer compared to the needs of the department. | *Overall evaluation* |
